# Targeting TARDBP to Restore Colonic Barrier Integrity in Ulcerative Colitis via NFATC1 mRNA Destabilization

**DOI:** 10.5152/tjg.2026.25318

**Published:** 2026-06-23

**Authors:** Yin Jiang, JingLin Gao, YaLi Huang, XingHui Song, GuangTing Li, Yuan Jiang, XiuHua Liu, DaGui Chen

**Affiliations:** 1 Department of Rheumatology and Immunology, Liuzhou Key Laboratory of Prevention and Treatment of Rheumatic Diseases, The Fourth Affiliated Hospital of Guangxi Medical University (Liuzhou Worker's Hospital), Liuzhou, China; 2 Department of Cardiology, Sun Yat-Sen Memorial Hospital of Sun Yat-Sen University, Guangzhou, China; 3 Guangzhou Key Laboratory of Molecular Mechanisms of Major Cardiovascular Disease, Sun Yat-Sen Memorial Hospital of Sun Yat-Sen University, Guangzhou, China; 4 Guangdong Province Key Laboratory of Arrhythmia and Electrophysiology, Sun Yat-Sen Memorial Hospital of Sun Yat-Sen University, Guangzhou, China; 5 Department of Oncology, Liuzhou Key Laboratory of Prevention and Treatment of Rheumatic Diseases, The Fourth Affiliated Hospital of Guangxi Medical University (Liuzhou Worker's Hospital), Liuzhou, China

**Keywords:** Cytoplasmic 1, dextran sulfate sodium, nuclear factor of activated T-cells, ulcerative colitis, TAR DNA-binding protein

## Abstract

**Background/Aims:**

Ulcerative colitis (UC) is a chronic inflammatory bowel disease characterized by limited understanding of post-transcriptional mechanisms governing intestinal barrier integrity. This study investigated the role of NFATC1 in modulating barrier function during colitis and identified RNA-binding proteins regulating its expression.

**Materials and Methods:**

An experimental model of UC was established in mice using dextran sulfate sodium (DSS). Adeno-associated virus vectors were used for in vivo knockdown of *NFATC1* or overexpression of *TARDBP*. Colonic pathology was evaluated by histologic analysis and terminal deoxynucleotidyl transferase dUTP nick end labeling (TUNEL) assays for apoptosis. Inflammatory cytokines, oxidative stress markers, and intestinal permeability were quantified using enzyme-linked immunosorbent assay (ELISA) and commercial kits. The expression levels of *NFATC1*, *TARDBP*, *TLR4*/p-p65, Zonula occludens-1 (*ZO-1)*, and *occludin* were assessed by reverse transcription quantitative polymerase chain reaction (RT-qPCR), western blotting, and immunofluorescence staining. The molecular interaction between TARDBP and NFATC1 was investigated using coimmunoprecipitation, actinomycin D chase assays, and RNA immunoprecipitation.

**Results:**

DSS administration impaired colonic barrier integrity in mice and was associated with increased NFATC1 and decreased TARDBP expression. Notably, *NFATC1* knockdown or *TARDBP* overexpression independently ameliorated DSS-induced colonic barrier damage. In contrast, the protective effects of TARDBP overexpression were abrogated by simultaneous *NFATC1* overexpression. Mechanistically, TARDBP directly bound to *NFATC1* mRNA, thereby promoting its degradation and reducing its stability rather than interacting at the protein level.

**Conclusion:**

This study identified a novel post-transcriptional regulatory mechanism by which TARDBP attenuates colonic inflammation through destabilization of *NFATC1* mRNA. These findings highlight the TARDBP-NFATC1 axis as a potential therapeutic target for restoring intestinal barrier function in UC.

**Main Points**

- NFATC1 knockdown ameliorates dextran sulfate sodium (DSS)-induced impairment of colonic barrier function.
- TARDBP targets and reduces NFATC1 mRNA stability.
- TARDBP overexpression ameliorates DSS-induced impairment of colonic barrier function.

## Introduction

Ulcerative colitis (UC) is a chronic inflammatory bowel disease characterized by recurrent episodes of inflammation confined to the mucosal layer of the colon and rectum.^1^ The pathogenesis of UC is driven by a multifaceted interaction of genetic factors, environmental stimuli, immune system dysregulation, and gut microbiota disturbances.^2^ Clinical manifestations of UC include rectal bleeding, diarrhea, and abdominal pain, often accompanied by extraintestinal complications affecting the skin, joints, and hepatobiliary system.^3^ Current therapeutic strategies include pharmacological agents (e.g., 5-aminosalicylic acid, corticosteroids, immunomodulators, and biologics), surgical intervention, and dietary management.^4^^,^^5^ Despite their efficacy in symptom control, many patients require long-term therapy, which is associated with significant side effects and eventual loss of response, highlighting the need for novel therapeutic targets.^6^

NFATC1, a member of the nuclear factor of activated T-cell transcription factor family, is encoded by the NFATC1 gene located on human chromosome 18.^7^ Its highly conserved Rel homology domain confers DNA-binding activity, making it a critical regulator of T-cell activation, differentiation, and cytokine production.^8^^-^^10^ In T cells, NFATC1 cooperates with AP-1 family members to coactivate the transcription of key cytokine genes, such as interleukin-2 (IL-2), which are essential for T-cell proliferation and immune responses.^11^ Accumulating evidence has implicated NFATC1 in the pathogenesis of UC. For instance, NFATC1-positive stem-like cells have been shown to contribute to colonic epithelial regeneration in dextran sulfate sodium (DSS)-induced experimental colitis.^12^ Furthermore, increased nuclear translocation of NFATC1 in T cells is a feature of colitis models.^13^^,^^14^ However, the precise impact of NFATC1 on intestinal barrier function and the upstream molecular mechanisms regulating its expression remain incompletely understood.

RNA-binding proteins (RBPs) are key regulators of RNA metabolism and control processes such as splicing, stability, and translation.^15^ The role of RBPs in regulating NFATC1 expression, particularly in the context of intestinal inflammation, remains largely unexplored. This study aimed to investigate the functional role of NFATC1 in DSS-induced colonic barrier dysfunction and to identify RBPs that may post-transcriptionally regulate its expression. It was hypothesized that a specific RBP modulates NFATC1 levels, thereby influencing the severity of colitis. Elucidating this regulatory axis may provide a scientific basis for the development of novel therapeutic strategies for UC, potentially improving treatment efficacy and reducing reliance on long-term systemic therapy.

## Materials and Methods

### Establishment of Ulcerative Colitis Mouse Model

All animal procedures were approved by the Animal Ethics Committee of The Fourth Affiliated Hospital of Guangxi Medical University (Approval No: 202306GX-37; Date: July 1, 2023). Six-week-old male C57BL/6J mice (21 ± 2 g, specific pathogen free (SPF) grade) were obtained from Spife Biotechnology (Beijing, China) and housed under controlled conditions (24°C ± 2°C, 50% ± 10% humidity, 12-hour light/dark cycle) with ad libitum food and sterile water. After 1 week of acclimatization, the mice were randomly assigned to 6 groups (n = 8 per group): control, DSS, adeno-associated virus (AAV)-NC, AAV-oe-*NFATC1*, AAV-oe-*TARDBP*, and AAV-oe-*NFATC1* + AAV-sh-*TARDBP*. Except for the control group, colitis was induced by the administration of 3% DSS (molecular weight 36 000-50 000 Da; MP Biomedicals, Irvine, CA, USA) in drinking water for 7 days.^16^ This concentration was selected based on preliminary experiments and established literature,^16^ as it reliably induces a moderate and reproducible colitis phenotype while maintaining a low mortality rate. Body weight was recorded daily. On day 8, mice were anesthetized with pentobarbital sodium (50 mg/kg, i.p.). Blood samples were collected via retro-orbital puncture and centrifuged at 3000 × g for 15 minutes at 4°C), and serum was stored at −80°C. After cervical dislocation, the spleen and colon were harvested; weighed, and fixed in 4% paraformaldehyde or snap-frozen. This study did not use AI and/or LLM tools during the preparation of this manuscript.

### Adeno-Associated Virus Vector Administration

AAV serotype 9 (AAV9) vectors encoding sh-*NFATC1*, oe-*NFATC1*, oe-*TARDBP*, or negative controls were purchased from PackGene (Guangzhou, China). In vivo gene delivery was performed as previously described.^17^ Briefly, mice were lightly anesthetized with isoflurane, and a flexible 24-gauge catheter was inserted 4 cm into the anus. An AAV vector suspension (4 × 10^10^ VG in 100 µL sterile phosphate-buffered saline (PBS)) was slowly administered into the colon. Mice were then maintained in a head-down position for 1 minute to facilitate distribution throughout the colon. AAV administration was performed 3 days before the initiation of DSS treatment (Supplementary Figure 1). This pretreatment schedule was selected to allow sufficient time for robust transgene expression in the colonic epithelium before the onset of DSS-induced injury, ensuring that the genetic intervention was functionally active at the initiation of the inflammatory challenge.

### Disease Activity Index Scoring

The Disease Activity Index (DAI) was assessed daily on the basis of combined scores for body weight loss, stool consistency, and rectal bleeding. Body weight loss was scored as follows: 0 (no loss), 1 (0%-5%), 2 (5%-10%), 3 (10%-15%), and 4 (>15%). Stool consistency was scored as 0 (normal), 2 (loose stools), and 4 (diarrhea). Rectal bleeding was scored as 0 (normal), 2 (occult blood positive), and 4 (severe bleeding). The final DAI score was calculated as the mean of the 3 parameters.

### Hematoxylin and Eosin Staining

Colon tissues were fixed in 4% paraformaldehyde, dehydrated through a graded ethanol series (70%, 80%, 95%, and 100%), cleared in xylene (Sigma-Aldrich, St. Louis, MO, USA), and embedded in paraffin (Leica Biosystems, Wetzlar, Germany). Paraffin-embedded tissues were sectioned at a thickness of 4 μm. The sections were deparaffinized in xylene and rehydrated through a graded ethanol series. Hematoxylin and eosin staining was performed using a commercial kit (Sangon, Shanghai, China). Briefly, the sections were stained with hematoxylin for 5 minutes, differentiated in 1% acid alcohol, rinsed with running tap water, and counterstained with eosin for 1 minute. The sections were then dehydrated, cleared, and mounted with neutral balsam (Solarbio, Beijing, China). Images were captured using a Nikon Eclipse E100 light microscope equipped with a Nikon DS-U3 imaging system (Nikon, Tokyo, Japan) and analyzed using CaseViewer 2.4 software (3DHISTECH, Hungary).

### TUNEL Staining

Apoptosis in colonic tissue was detected using the One-Step TUNEL Apoptosis Assay Kit (Roche, Shanghai, China; APT110). Paraffin-embedded sections were deparaffinized, rehydrated, and permeabilized with proteinase K (20 µg/mL in PBS; Sigma-Aldrich) for 20 minutes. Sections were then incubated for 1 hour at 37°C in a humidified chamber with a mixture of terminal deoxynucleotidyl transferase (TdT) and fluorescently labeled dUTP according to the manufacturer's protocol. For positive controls, sections were pretreated with DNase I for 10 minutes at 25°C. For negative controls, TdT enzyme was omitted. Nuclei were counterstained with DAPI (1 µg/mL; Sigma-Aldrich; Merck KGaA, Darmstadt, Germany) for 10 minutes at room temperature, and sections were mounted using an antifade mounting medium (Vector Laboratories, Burlingame, CA, USA). Images were acquired using a Carl Zeiss LSM700 confocal microscope (Zeiss, Oberkochen, Germany).

### ELISA

Colon tissue samples (approximately 50 mg) were homogenized in 500 µL of ice-cold PBS containing a protease inhibitor cocktail (Roche, Basel, Switzerland). The homogenates were centrifuged at 12 000 × g for 15 minutes at 4°C, and the supernatants were collected. Concentrations of tumor necrosis factor-α (TNF-α), IL-1β, and IL-6 in colonic tissue homogenates were quantified using ELISA kits from Thermo Fisher Scientific (Waltham, MA, USA; Cat. No. 88-7324, 88-7013, and 88-7064, respectively). Serum levels of lipopolysaccharide (LPS) and diamine oxidase (DAO) were measured using ELISA kits from Cloud-Clone (Wuhan, China). All assays were performed in duplicate.

### Oxidative Stress Markers

Malondialdehyde (MDA), superoxide dismutase (SOD), and catalase (CAT) in colon tissue homogenates were measured using commercial assay kits (Cat. No. A003-1 for MDA, A001-3 for SOD, and A007-1 for CAT; Jiancheng Bioengineering Institute, Nanjing, China). Tissue homogenates were prepared according to the manufacturer’s instructions.

### RT-qPCR

Total RNA was isolated from tissues or cells using TRIzol reagent (Invitrogen, Carlsbad, CA, USA; 15596026) following the manufacturer’s instructions. Briefly, samples were homogenized, and phase separation was performed using chloroform (Sigma-Aldrich), followed by RNA precipitation with isopropanol and washing with 75% ethanol. The RNA pellet was resuspended in nuclease-free water. RNA concentration and purity were determined using the NanoDrop 2000c spectrophotometer (Thermo Fisher), and RNA integrity was assessed using the Agilent 2100 Bioanalyzer (Agilent Technologies). Samples with an RNA Integrity Number >7.0 were considered acceptable. Complementary DNA (cDNA) was synthesized using HiScript III RT SuperMix for qPCR (+gDNA wiper) (Vazyme; R323-01) on a T100 Thermal Cycler (Bio-Rad, Hercules, CA, USA). Quantitative PCR was performed using Universal SYBR qPCR Master Mix (Vazyme, Nanjing, China; Q511-02) on a CFX96 Real-Time PCR Detection System (Bio-Rad). Relative gene expression was calculated using the 2^−ΔΔCt^ method with *GAPDH* as the internal reference. Primers were synthesized by Sangon Biotech (Shanghai, China) and are listed in Supplementary Table 1.

### Western Blot

Colon tissues were homogenized on ice in radioimmunoprecipitation assay (RIPA) buffer (Beyotime, Shanghai, China) containing a protease inhibitor cocktail (Roche) using a tissue grinder, followed by brief sonication. The lysates were centrifuged at 14 000 × *g* for 20 minutes at 4°C, and the supernatants were collected for protein analysis. Protein concentrations were quantified using a bicinchoninic acid (BCA) protein assay kit (Beyotime). Equal amounts of protein (30 μg) were separated by 8%-13% sodium dodecyl sulfate-polyacrylamide gel electrophoresis (SDS-PAGE) and transferred onto polyvinylidene difluoride membranes (Millipore, Billerica, MA, USA) using a wet transfer system (Bio-Rad) at 100 V for 90 minutes. Membranes were blocked with 5% nonfat milk in tris-buffered saline containing 0.1% Tween 20 (TBST) for 1 hour at room temperature and incubated overnight at 4°C with primary antibodies (1 : 1000). After 3 washes with TBST, membranes were incubated with horseradish peroxidase (HRP)-conjugated secondary antibody (ab6728, Abcam, Cambridge, UK, 1 : 5000) for 1 hour. Protein bands were visualized using ECL Prime detection reagent (GE Healthcare, Chicago, IL, USA) and captured using a da Vinci chemiluminescence imaging system (CoreBio, Seoul, Korea). Primary antibodies included glyceraldehyde-3-phosphate dehydrogenase (GAPDH) (2118, CST), TLR4 (19811-1-AP, Proteintech), p-p65 (3033, CST), p65 (8242, CST), TARDBP (10782-2-AP, Proteintech), and NFATC1 (8032, CST).

### Immunofluorescence Staining

Paraffin sections were deparaffinized, rehydrated, and subjected to antigen retrieval by boiling in 10 mM sodium citrate buffer (pH 6.0) for 20 minutes. Sections were then blocked with 0.5% bovine serum albumin (Sigma-Aldrich) for 30 minutes at room temperature, followed by an overnight incubation at 4°C with primary antibodies against ZO-1 (ab221547, Abcam; 1 : 200 dilution) and occludin (ab216327, Abcam; 1 : 200 dilution). Sections were subsequently washed 3 times with TBS and incubated with Alexa Fluor 488- and 594-tagged anti-rabbit IgG (ab150077 and ab150080, Abcam; 1 : 500 dilution) for 1.5 hours at 37°C. After 3 additional washes with TBST, sections were mounted using ProLong Gold Antifade Mountant containing DAPI (Thermo Fisher Scientific). Images were acquired using a Nikon Eclipse C1 fluorescence microscope and Nikon DS-U3 imaging system (Nikon) and analyzed using Caseviewer 2.4 software (3DHISTECH Ltd, Budapest, Öv u. 3., Hungary).

### Cell Culture

The Caco-2 cell line was selected as a well-established model of the human intestinal epithelial barrier. These cells spontaneously differentiate into a polarized monolayer with tight junctions and a brush border, closely mimicking the functional characteristics of intestinal epithelial cells. Human intestinal epithelial Caco-2 cells (KeyGen Biotech, Nanjing, China) were cultured in Dulbecco's modified Eagle medium (Gibco, Grand Island, NY, USA) supplemented with 10% fetal bovine serum (Gibco), 100 U/mL penicillin, and 100 µg/mL streptomycin (Gibco) at 37°C in a humidified atmosphere containing 5% CO_2_.

### Cell Transfection

Small interfering RNAs (siRNAs) targeting *TARDBP* and a nontargeting control, as well as pcDNA3.1-based overexpression vectors for *TARDBP* and an empty vector control, were synthesized by GenePharma (Shanghai, China). Caco-2 cells were seeded to reach 60%-70% confluency on the day of transfection. Cells were transiently transfected using Lipofectamine 3000 (Invitrogen). Briefly, 50 nM of siRNA or 2.5 µg of plasmid DNA was used per well in a 6-well plate. Transfection efficiency was confirmed by RT-qPCR and western blot analysis 48 hours after transfection.

### Actinomycin D Assay

To assess the effect of *TARDBP* on *NFATC1* mRNA stability, Caco-2 cells transfected with TARDBP-overexpressing or control vectors were treated with 5 µg/mL actinomycin D (Sigma-Aldrich, Cat. No. A9415; dissolved in DMSO) to inhibit de novo transcription. Total RNA was extracted at specified time points (0, 2, 4, and 6 hours), and the remaining levels of *NFATC1* mRNA were quantified by RT-qPCR.

### Coimmunoprecipitation

Caco-2 cells were lysed using IP lysis buffer (Beyotime, P0013B). Protein A/G magnetic beads (Thermo Fisher Scientific, Cat. No. 88802) were used to pre-clear cell lysates for 1 hour at 4°C. Following clearance, the lysates were incubated overnight at 4°C with either 5 μg of anti-TARDBP antibody or control IgG antibody. Protein A/G magnetic beads were added and incubated for 2 hours at 4°C to capture the immunocomplexes. After centrifugation, the sediment was collected and washed 3 times with ice-cold IP lysis buffer. Proteins were eluted from the beads through 1× SDS-PAGE and heated for 10 minutes. Samples were subsequently subjected to western blot analysis.

### RNA Immunoprecipitation

RNA immunoprecipitation (RIP) assays were performed using the EZ-Magna RIP Kit (Millipore) according to the manufacturer’s protocol. Briefly, Caco-2 cell lysates were incubated with magnetic beads conjugated to 5 µg anti-TARDBP antibody or control anti-IgG antibody. After incubation and washing, coprecipitated RNA was isolated from the immunoprecipitated complexes. The purified RNA was then analyzed by RT-qPCR to quantify enrichment of *NFATC1* mRNA. Results were normalized to input RNA levels.

### Data Analysis

Statistical analyses were performed using GraphPad Prism 9.0 (GraphPad Software; San Diego, California, USA). All experiments were performed with at least 3 biological replicates. Data are presented as mean ± SD. Comparisons between 2 groups were performed using an unpaired 2-tailed Student’s *t*-test. Comparisons among multiple groups were performed using 1-way analysis of variance followed by Tukey's multiple comparisons post-hoc test. A *P*-value < .05 was considered statistically significant.

## Results

### Dextran Sulfate Sodium Induces Colitis and Upregulates *NFATC1* Expression in Mice

**Figure 1.**
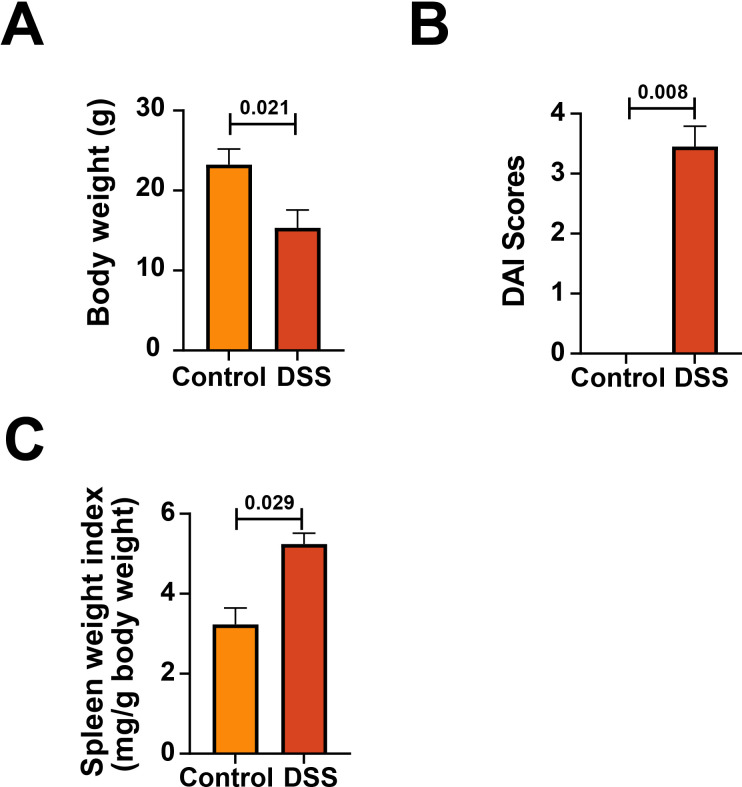
Dextran sulfate sodium (DSS) induces colonic barrier dysfunction and upregulates *NFATC1* expression in mice. Treatment with DSS was used to induce ulcerative colitis in mice. (A) Changes in body weight of mice. (B) Time-dependent effects of DSS treatment on colonic dysfunction in mice were assessed by calculating the Disease Activity Index. (C) Effects of DSS treatment on systemic immune activation in mice were assessed by measuring the spleen weights. Data are expressed as mean ± SD (n = 8). *P < .05.

Administration of 3% DSS for 7 days successfully induced experimental colitis in mice. Compared with controls, DSS-treated mice exhibited significant body weight loss (Figure 1A) and progressively increased DAI scores (Figure 1B), accompanied by splenomegaly indicative of systemic immune activation (Figure 1C). Histological examination revealed severe colonic mucosal injury, including epithelial erosion, crypt destruction, and extensive inflammatory cell infiltration (Supplementary Figure 2A). TUNEL staining demonstrated increased epithelial apoptosis (Supplementary Figure 2B). ELISA analysis demonstrated significantly increased levels of the proinflammatory cytokines TNF-α, IL-1β, and IL-6 in colon tissues (Supplementary Figure 2C). DSS exposure also aggravated oxidative stress, as evidenced by increased MDA levels and reduced SOD and CAT activities (Supplementary Figure 2D). Intestinal barrier dysfunction was confirmed by elevated serum LPS and DAO levels (Supplementary Figure 2E) and disrupted expression of tight junction proteins ZO-1 and occludin (Supplementary Figure 2F). Moreover, DSS activated the *TLR4*/NF-κB pathway, as demonstrated by increased expression of *TLR4* and phosphorylated p65 (Supplementary Figure 2G). Notably, *NFATC1* mRNA and *NFATC1* protein levels were significantly upregulated (Supplementary Figure 2H, I).

### *NFATC1* Knockdown Ameliorates Dextran Sulfate Sodium-Induced Colonic Barrier Impairment

**Figure 2.**
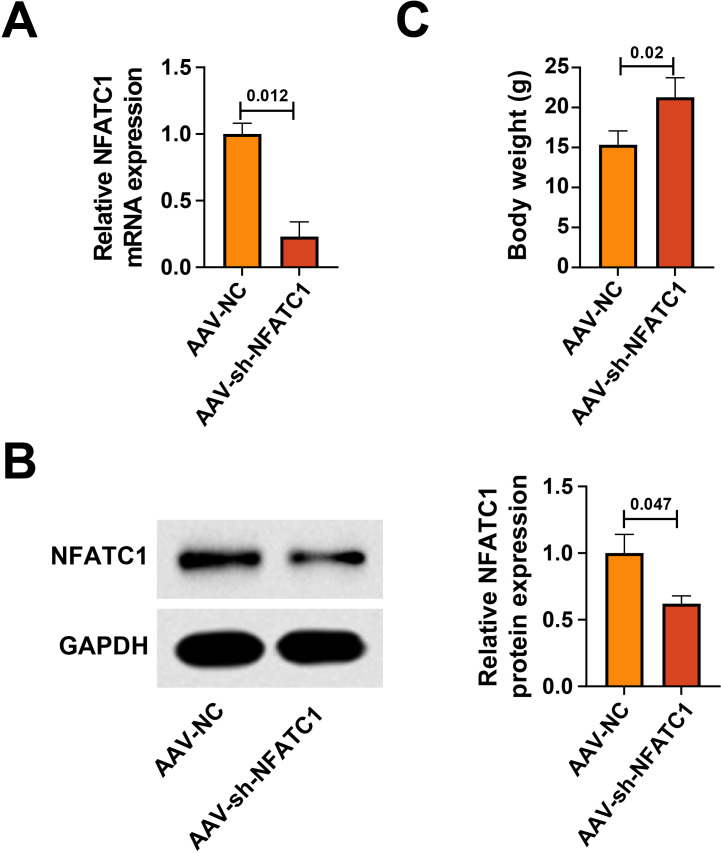
Knockdown of *NFATC1* ameliorates dextran sulfate sodium (DSS)-induced colonic barrier dysfunction. DSS mice were pretreated using AAV-sh-*NFATC1*. (A and B) mRNA and protein expression of *NFATC1* were detected by RT-qPCR and western blot in the colon tissues of the mice, respectively. (C) Changes in body weight in mice. Data are expressed as mean ± SD (n = 8). *P < .05.

To determine the functional role of *NFATC1* in colitis, mice were administered AAV-sh-*NFATC1* before DSS challenge. This treatment reduced colonic *NFATC1* mRNA and protein expression (Figure 2A, B). *NFATC1* knockdown significantly attenuated DSS-induced weight loss (Figure 2C), decreased the DAI, and reduced spleen weight (Supplementary Figure 3A, B). Histological analysis revealed improved colonic architecture with reduced inflammatory infiltration (Supplementary Figure 3C). Consistently, epithelial apoptosis and levels of proinflammatory cytokine were decreased (Supplementary Figure 3D, E). *NFATC1* silencing also alleviated oxidative stress, as evidenced by restoration of MDA, SOD, and CAT levels (Supplementary Figure 3F), and improved intestinal barrier integrity, as demonstrated by reduced serum LPS and DAO and preserved ZO-1 and occludin expression (Supplementary Figure 3G, H). Mechanistically, activation of *TLR4* and p-p65 was suppressed (Supplementary Figure 3I).

### *TARDBP* Directly Binds to and Destabilizes *NFATC1* mRNA

**Figure 3.**
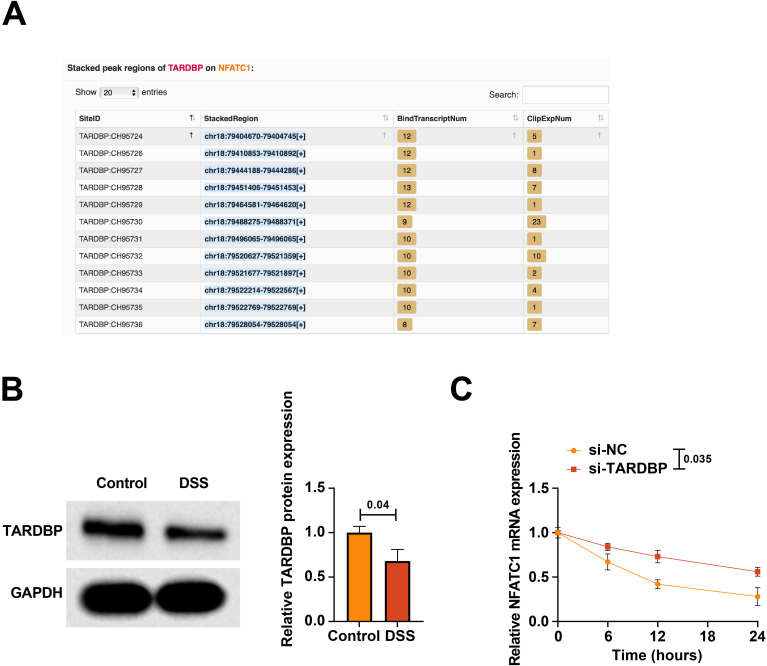
RNA-binding protein TARDBP targets to reduce NFATC1 mRNA stability. (A) The starBase database showing multiple potential binding sites for TARDBP and NFATC1. (B) Western blot analysis of TARDBP expression in colon tissues of DSS-treated mice. (C) Actinomycin D assay assessing the effect of knockdown of TARDBP on NFATC1 mRNA stability. Data are expressed as mean ± SD (n = 3). *P < .05.

To identify RBPs regulating *NFATC1*, bioinformatic analysis using the ENCORI/starBase database predicted *TARDBP*-binding sites on the *NFATC1* transcript (Figure 3A). *TARDBP* protein expression was significantly reduced in the colons of DSS-treated mice and showed an inverse association with *NFATC1* levels (Figure 3B). Actinomycin D chase assays in Caco-2 cells demonstrated that TARDBP knockdown significantly prolonged the half-life of NFATC1 mRNA, indicating reduced transcript degradation (Figure 3C). RIP assays further confirmed direct binding between TARDBP and NFATC1 mRNA (Supplementary Figure 4A), whereas coimmunoprecipitation detected no protein-level interaction (Supplementary Figure 4B). Consistently, TARDBP knockdown increased NFATC1 protein expression, whereas TARDBP overexpression decreased it (Supplementary Figure 4C). These findings indicated that TARDBP destabilizes NFATC1 mRNA through post-transcriptional regulation.

### Overexpression of *TARDBP* Does Not Affect Colonic Barrier Function in Normal Mice

The study evaluated whether *TARDBP* overexpression affects intestinal homeostasis in normal mice. AAV-oe-*TARDBP* significantly increased *TARDBP* mRNA levels (Supplementary Figure 5A). However, histological examination showed no structural alterations in the colon (Supplementary Figure 5B), and the proportion of TUNEL-positive cells remained unchanged (Supplementary Figure 5C). Inflammatory cytokines, including TNF-α, IL-1β, and IL-6, were also unaffected (Supplementary Figure 5D). Oxidative stress markers (MDA, SOD, and CAT) showed no significant differences between groups (Supplementary Figure 5E). Consistently, intestinal barrier function indicators, serum LPS and DAO, were unchanged (Supplementary Figure 5F). Moreover, TARDBP overexpression did not alter the expression of tight junction proteins (ZO-1 and occludin) (Supplementary Figure 5G) or the expression of *TLR4* and p-p65 (Supplementary Figure 5H). Together, these findings indicated that *TARDBP* overexpression does not disturb normal intestinal homeostasis.

### *TARDBP* Overexpression Ameliorates Dextran Sulfate Sodium–Induced Colonic Barrier Impairment

**Figure 4.**
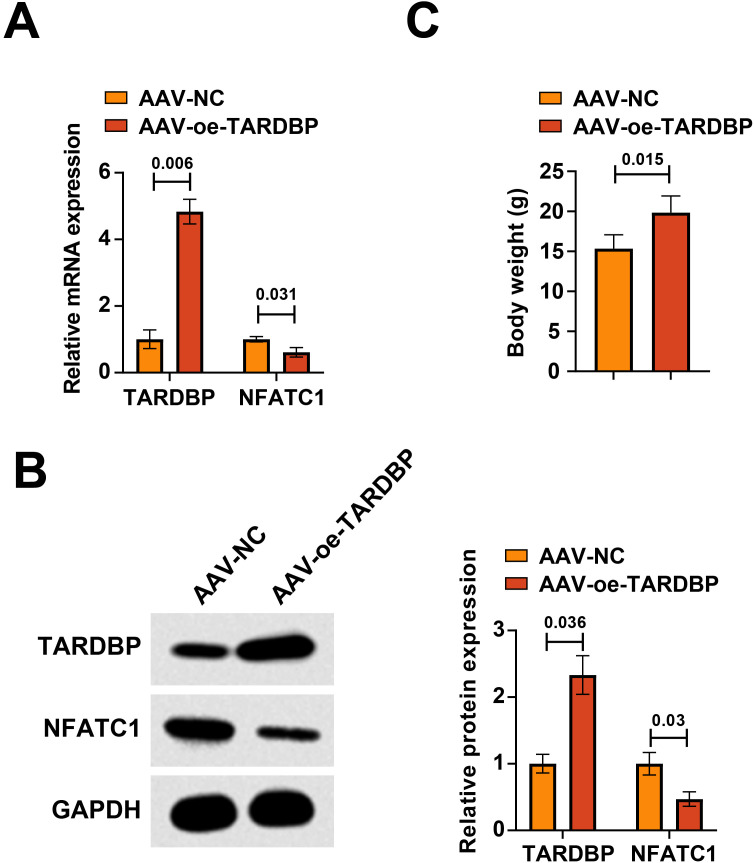
Overexpression of *TARDBP* ameliorates dextran sulfate sodium (DSS)–induced colonic barrier dysfunction. DSS-treated mice were pretreated with AAV-mediated *TARDBP* overexpression. (A and B) mRNA and protein expression of *TARDBP* and *NFATC1* in colon tissues of the mice were detected by RT-qPCR and western blot, respectively. (C) Changes in body weight of mice. Data are expressed as mean ± SD (n = 8). *P < .05.

To evaluate the therapeutic potential of *TARDBP*, AAV-oe-*TARDBP* was administered before DSS induction. *TARDBP* overexpression markedly increased *TARDBP* levels while reducing *NFATC1* expression in the colon (Figure 4A, B). Functionally, *TARDBP* significantly alleviated DSS-induced weight loss (Figure 4C), DAI scores, and splenomegaly (Supplementary Figure 6A, B). Histologic analysis demonstrated reduced mucosal injury, inflammatory infiltration, and epithelial apoptosis (Supplementary Figure 6C, D). Consistently, proinflammatory cytokines and oxidative stress markers were markedly decreased (Supplementary Figure 6E, F). *TARDBP* also improved intestinal barrier integrity, as evidenced by reduced serum LPS and DAO levels and preserved the expression of ZO-1 and occludin (Supplementary Figure 6G, H). These protective effects were accompanied by suppression of TLR4 and p-p65 signaling (Supplementary Figure 6I).

### The Protective Effect of *TARDBP* Is Mediated Through *NFATC1* Downregulation

**Figure 5.**
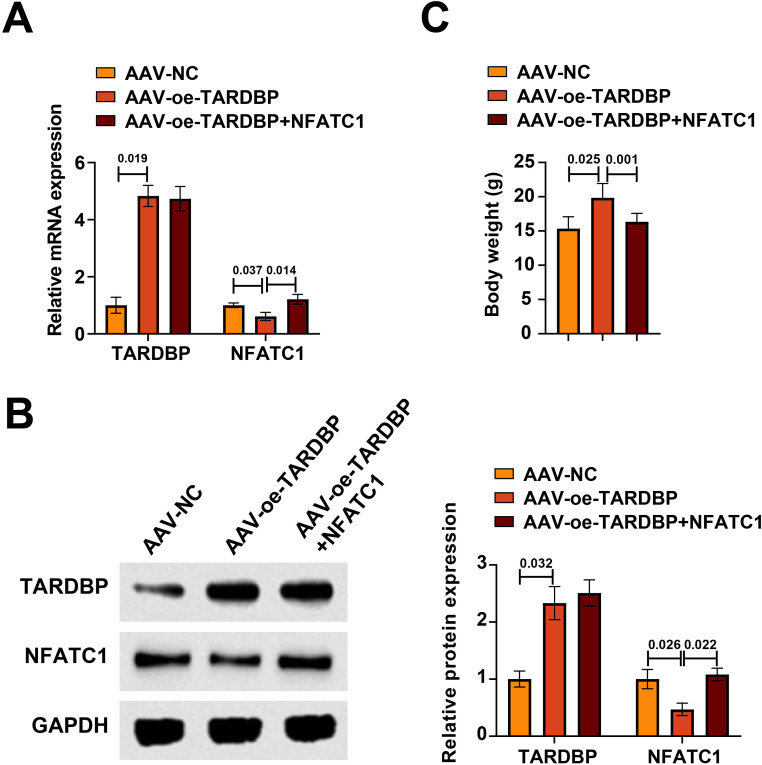
*TARDBP* destabilizes *NFATC1* mRNA to ameliorate dextran sulfate sodium (DSS)-induced impairment of colonic barrier function. AAV-mediated overexpression of *TARDBP* and overexpression of *NFATC1* strategy for preconditioning of DSS-treated mice. (A-B) mRNA and protein expression of *TARDBP* and *NFATC1* were detected by RT-qPCR and western blot in the colon tissues of the mice, respectively. (C) Changes in body weight of mice. Data are expressed as mean ± SD (n = 8). *P < .05.

To determine whether *TARDBP*-mediated protection depends on *NFATC1* regulation, rescue experiments were performed by coadministering AAV-oe-*TARDBP* and AAV-oe-*NFATC1* before DSS treatment. *TARDBP* overexpression increased *TARDBP* levels and suppressed endogenous *NFATC1*, whereas AAV-oe-*NFATC1* restored *NFATC1* expression without affecting TARDBP (Figure 5A, B). Importantly, *NFATC1* re-expression reversed the protective effects of *TARDBP*, including attenuation of body weight loss (Figure 5C), reductions in DAI score and spleen weight, preservation of colonic architecture, and decreased apoptosis. Likewise, the anti-inflammatory, antioxidative, barrier-protective, and *TLR4*/p-p65 inhibitory effects of *TARDBP* were abolished, indicating that *TARDBP* alleviates DSS-induced colitis primarily through *NFATC1* suppression.

## Discussion

This study identified a novel post-transcriptional regulatory mechanism in UC, demonstrating that the RBP *TARDBP* suppresses intestinal inflammation by destabilizing *NFATC1* mRNA. Restoration of *TARDBP* alleviated DSS-induced colitis and inhibited NFATC1-mediated inflammatory signaling, highlighting the *TARDBP*–*NFATC1* axis as a potential regulatory pathway and therapeutic target.

*NFATC1* is a well-established transcription factor involved in T-cell activation and immune regulation.^18^ Consistent with previous studies linking *NFATC1* to proinflammatory responses,^19^^,^^20^ the results showed that *NFATC1* knockdown significantly alleviated disease severity in experimental colitis, supporting its pathogenic role in intestinal inflammation. However, the function of *NFATC1* appears to be context dependent. Emerging evidence suggests that *NFATC1* may also contribute to epithelial regeneration following mucosal injury.^12^ This dual role complicates the feasibility of systemic *NFATC1* inhibition, as direct suppression could potentially impair epithelial repair. Therefore, identifying upstream regulatory mechanisms that modulate *NFATC1* expression may provide a more balanced therapeutic approach.

In this context, the findings identify *TARDBP* as a key upstream regulator of *NFATC1*. This finding is particularly novel, as *TARDBP* has mostly been studied in neurodegenerative diseases such as amyotrophic lateral sclerosis,^21^ whereas its role in intestinal inflammation remains largely unexplored. In contrast to proinflammatory RBPs such as HuR, which stabilize cytokine transcripts including TNF-α,^22^ *TARDBP* appears to function as a negative regulator of inflammation. Mechanistic analyses, including coimmunoprecipitation and RIP assays, confirmed that *TARDBP* directly interacts with *NFATC1* mRNA and promotes its degradation. Importantly, rescue experiments demonstrated that reintroduction of *NFATC1* abolished the protective effects of *TARDBP* overexpression, further supporting the functional importance of this regulatory interaction.

This study has several limitations. First, the DSS-induced colitis model primarily reflects acute epithelial injury and innate immune activation and does not fully recapitulate the chronic and relapsing nature of human UC. Second, this study focused on a single RBP–mRNA interaction,^23^ whereas *TARDBP* likely regulates a broader set of transcripts involved in intestinal inflammation. Third, translating AAV-mediated gene delivery into clinical therapy remains challenging due to issues such as cell-type specificity, immunogenicity, and long-term safety. Future studies should validate these findings in human samples and patient-derived intestinal organoid systems. In addition, transcriptomic analyses may help identify additional *TARDBP* targets and explore potential interactions with other regulatory pathways, such as *NOTCH1* signaling^10^ or other RBPs including HuR.

In conclusion, this study elucidated a critical, previously unappreciated role of the RBP *TARDBP* in maintaining intestinal homeostasis. *TARDBP* functions as a post-transcriptional repressor of *NFATC1*, thereby attenuating intestinal barrier damage and inflammation in a preclinical model of colitis. These findings provide new insights into the pathogenesis of UC and suggest that modulation of the *TARDBP*-*NFATC1* axis may represent a promising strategy for future therapeutic development.

## Supplementary Materials

Supplementary Material

## Data Availability

The data that support the findings of this study are available on request from the corresponding author.
