# Supplementary material for: Targeting TARDBP to Restore Colonic Barrier Integrity in Ulcerative Colitis via NFATC1 mRNA Destabilization
Source: Turk J Gastroenterol. 2026 Jun 23;37(8):869–79. doi: 10.5152/tjg.2026.25318 (PMC13455517; doi:10.5152/tjg.2026.25318)
Supplement: Supplementary file 1 [file supplementary_material.pdf]

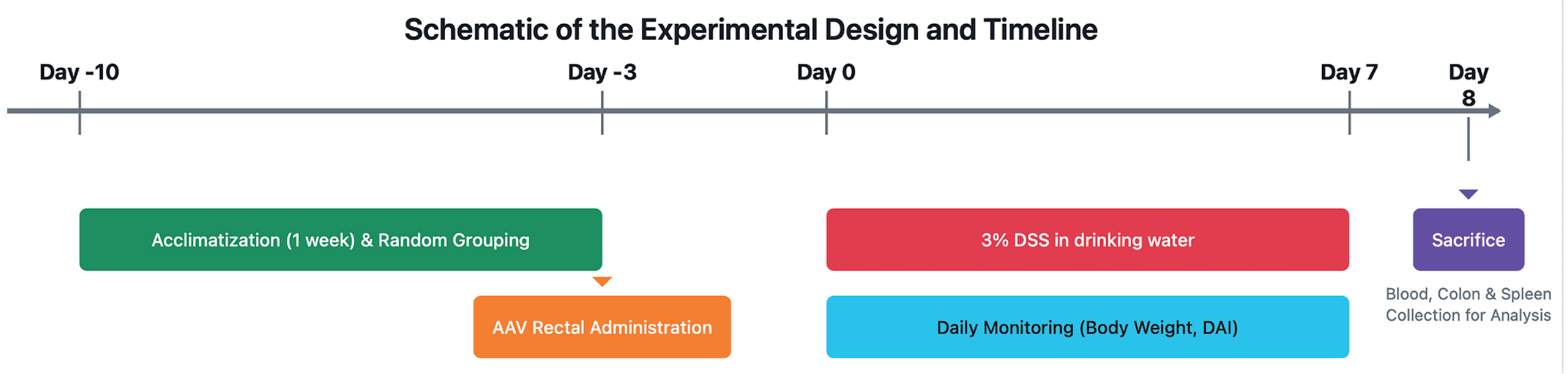

Supplementary Figure 1. Experimental flowchart

Supplementary Table 1. RT-qPCR primer sequences

Genes	Primer sequence
Nfatc1 (Mus musculus)	Forward: GGAGAGTCCGAGAATCGAGAT
	Reverse: TTGCAGCTAGGAAGTACGTCT
Tardbp (Mus musculus)	Forward: ATGAGAACGATGAACCCATTGAA
	Reverse: TGAGACACGGGATTCCGGTAG
GAPDH (Mus musculus)	Forward: AATGGATTTGGACGCATTGGT
	Reverse: TTTGCACTGGTACGTGTTGAT

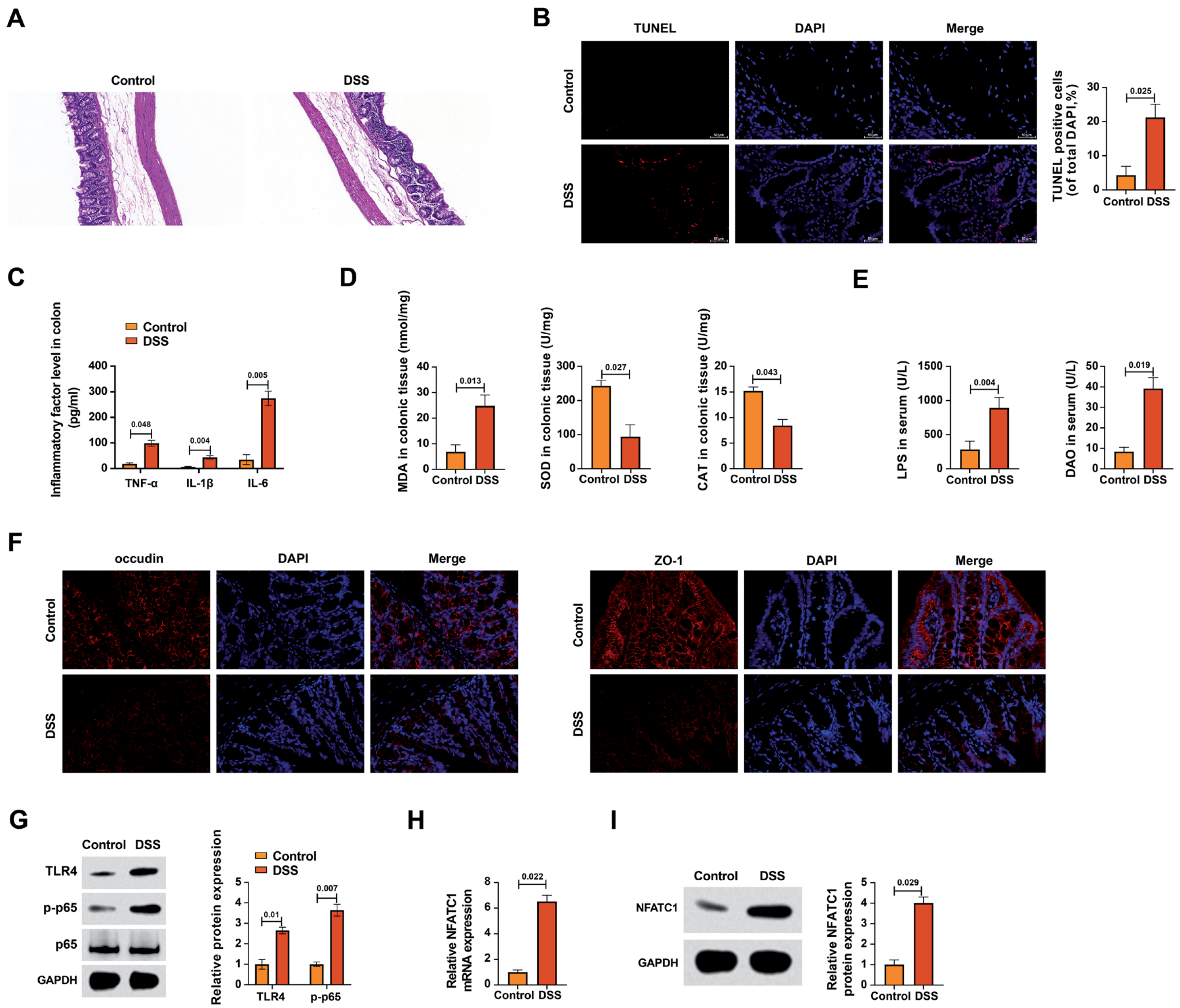

Supplementary Figure 2. DSS-induced pathological changes and molecular alterations in mouse colon. (A) Histological structure of epithelial cells of the colon of mice was assessed by HE staining. (B) Changes of apoptosis rate in the colon of mice were detected by TUNEL staining. (C) Levels of inflammatory factors TNF-α, IL-1β and IL-6 were detected by ELISA in the colon tissues of mice. (D) Oxidative damage was assessed by detecting the oxidative stress indicators MDA, SOD and CAT in the colon tissues of mice. (E) Intestinal permeability was assessed by serum LPS and DAO analysis in mice. (F) Changes in the expression of ZO-1 and occludin proteins in the colon tissues of mice were detected by IF staining. (G) Changes in TLR4 and p-p65 protein expression in the colon tissues of mice were detected by blot. (H-I) mRNA and protein expression of NFATC1 in the colon tissues of mice were detected by RT-qPCR and Western blot respectively. Data are expressed as mean ± SD (n = 8). *$P < 0.05$.

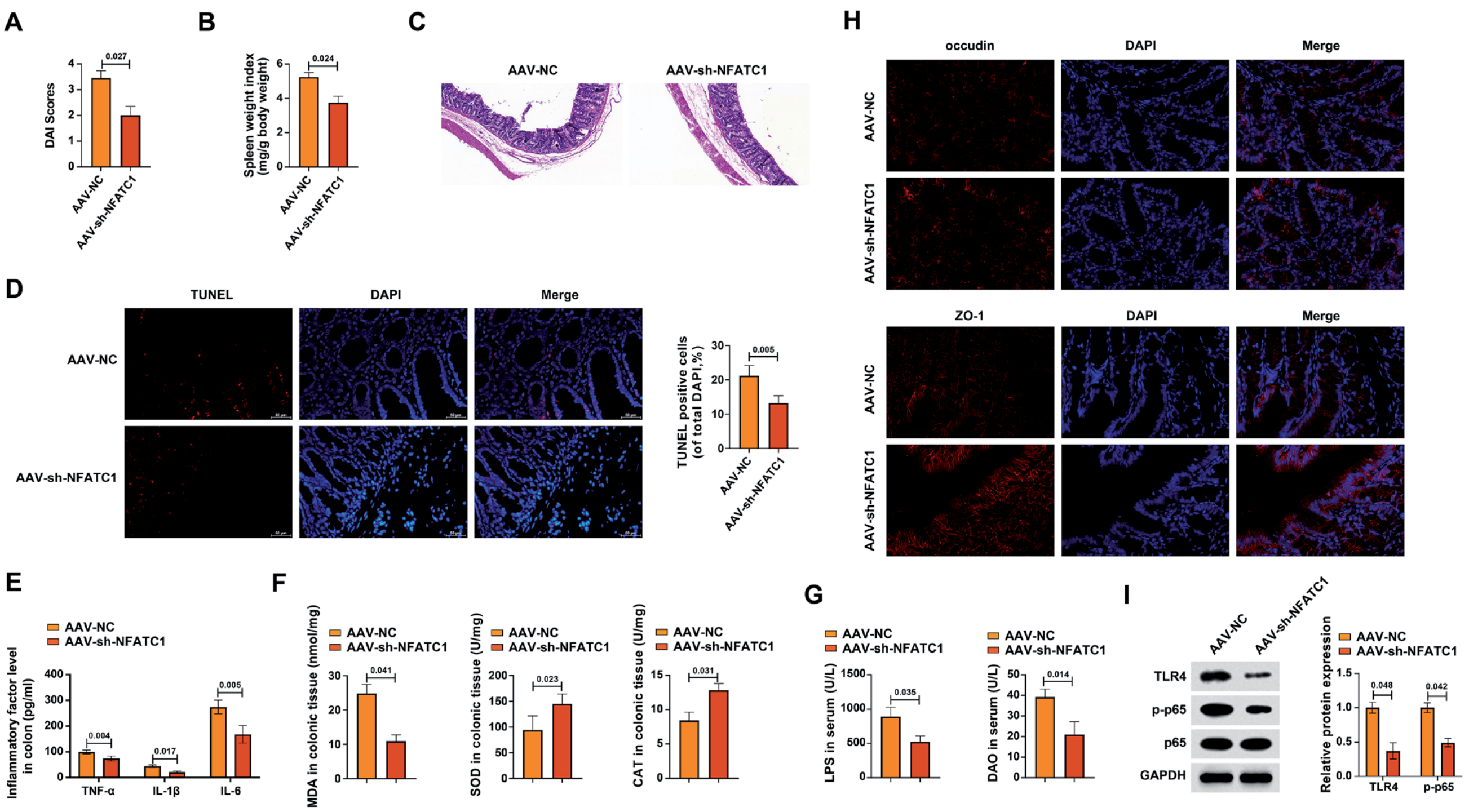

Supplementary Figure 3. NFATC1 knockdown improves DSS-induced colonic pathology. (A) Time-dependent effects of DSS treatment on functional impairment of the colon in mice were assessed by calculating DAI. (B) Effects of DSS treatment on systemic immune activation in mice were assessed by measuring the spleen weights. (C) Histological structure of epithelial cells of the colon of mice was assessed by HE staining. (D) Changes of apoptosis rate in the colon of mice were detected by TUNEL staining. (E) Levels of inflammatory factors TNF-α, IL-1β and IL-6 were detected by ELISA in the colon tissues of mice. (F) Oxidative damage was assessed by detecting the oxidative stress indicators MDA, SOD and CAT in the colon tissues of mice. (G) Intestinal permeability was assessed by serum LPS and DAO analysis in mice. (H) Changes in the expression of ZO-1 and occludin proteins in the colon tissues of mice were detected by IF staining. (I) Changes in TLR4 and p-p65 protein expression in the colon tissues of mice were detected by Western blot. Data are expressed as mean ± SD (n = 8). *$P < 0.05$.

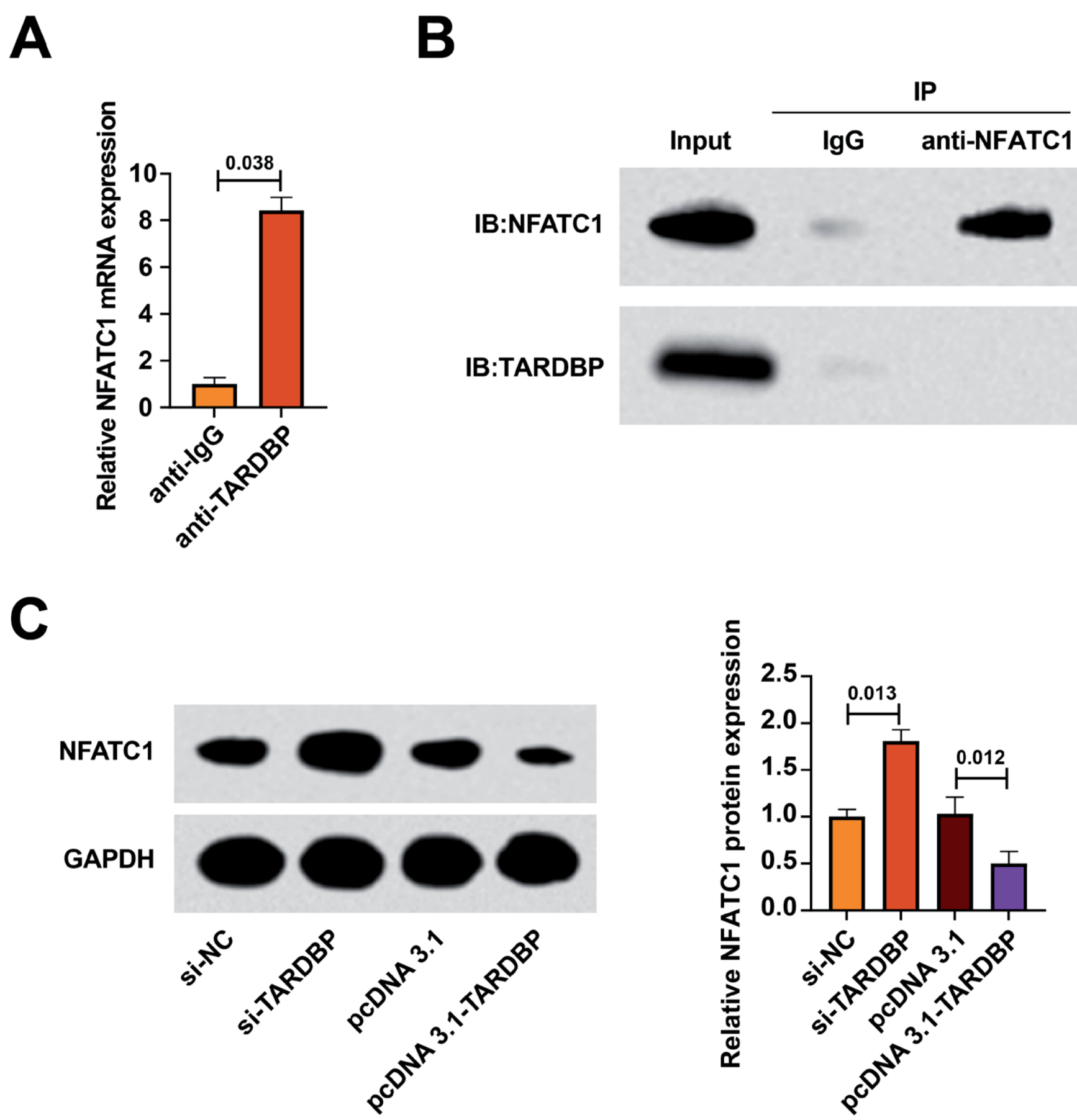

Supplementary Figure 4. TARDBP interacts with and regulates NFATC1. (A) RIP assay to detect the interaction between TARDBP and NFATC1. (B) CO-IP assay to detect the interaction between TARDBP and NFATC1. (C) Western blot to detect the effect of knockdown or overexpression of TARDBP on the protein expression of NFATC1. Data are expressed as mean ± SD (n = 3). *P < 0.05.

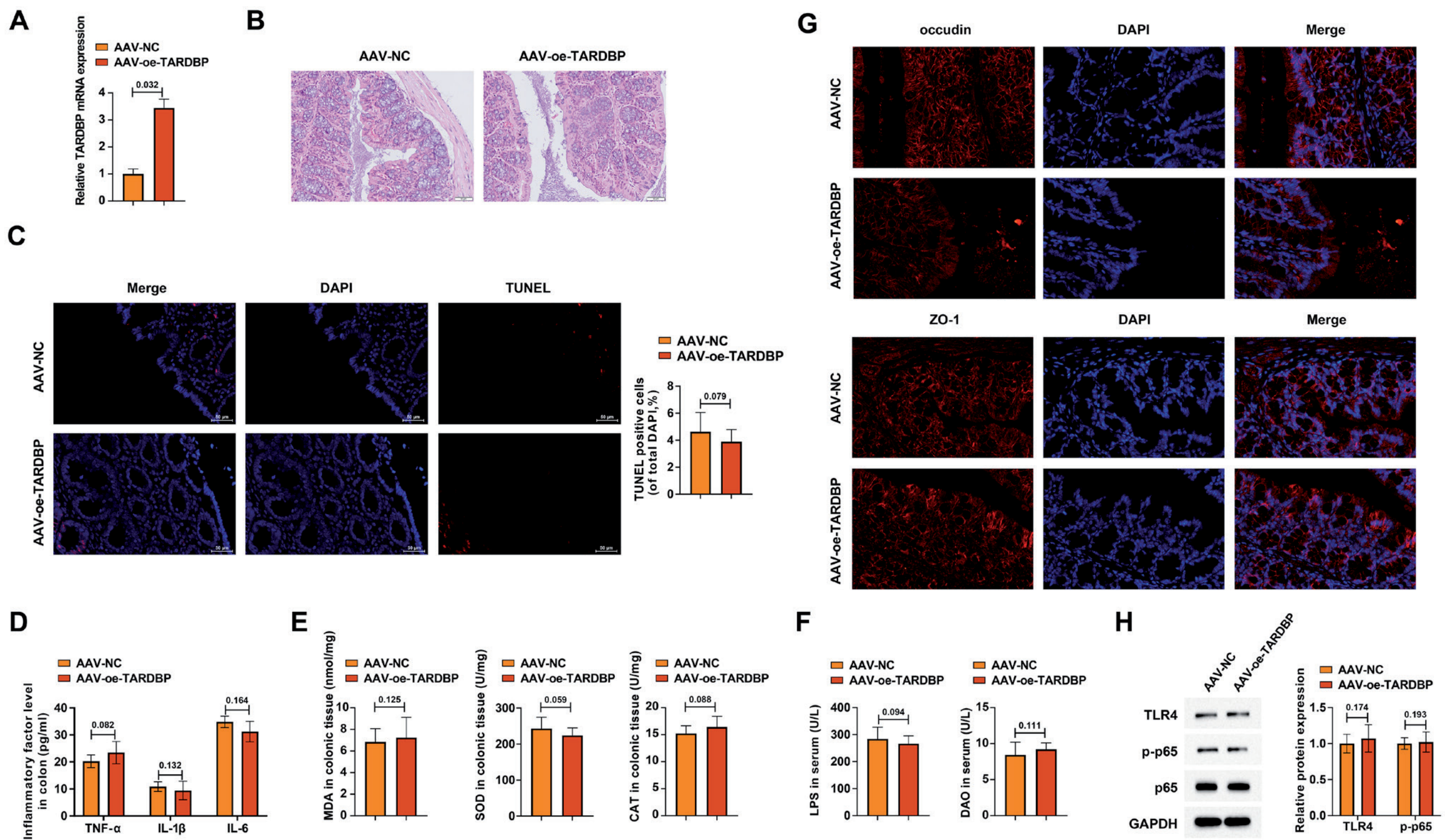

Supplementary Figure 5. Overexpression of TARDBP does not affect the colonic barrier function in normal mice. AAV was used to overexpress TARDBP in normal mice to assess its effects on colonic homeostasis. (A) The mRNA expression of TARDBP in the colon tissues of mice was detected by RT-qPCR. (B) Histological structure of the colon of mice was assessed by H&E staining. (C) Changes of apoptosis rate in the colon of mice were detected by TUNEL staining. (D) Levels of inflammatory factors TNF-α, IL-1β and IL-6 in the colon tissues of mice were detected by ELISA. (E) Oxidative damage was assessed by detecting the oxidative stress indicators MDA, SOD and CAT in the colon tissues of mice. (F) Intestinal permeability was assessed by serum LPS and DAO analysis in mice. (G) Changes in the expression of ZO-1 and occludin proteins in the colon tissues of mice were detected by IF staining. (H) Changes in TLR4 and p-p65 protein expression in the colon tissues of mice were detected by Western blot. Data are expressed as mean ± SD (n = 8). *$P < 0.05$.

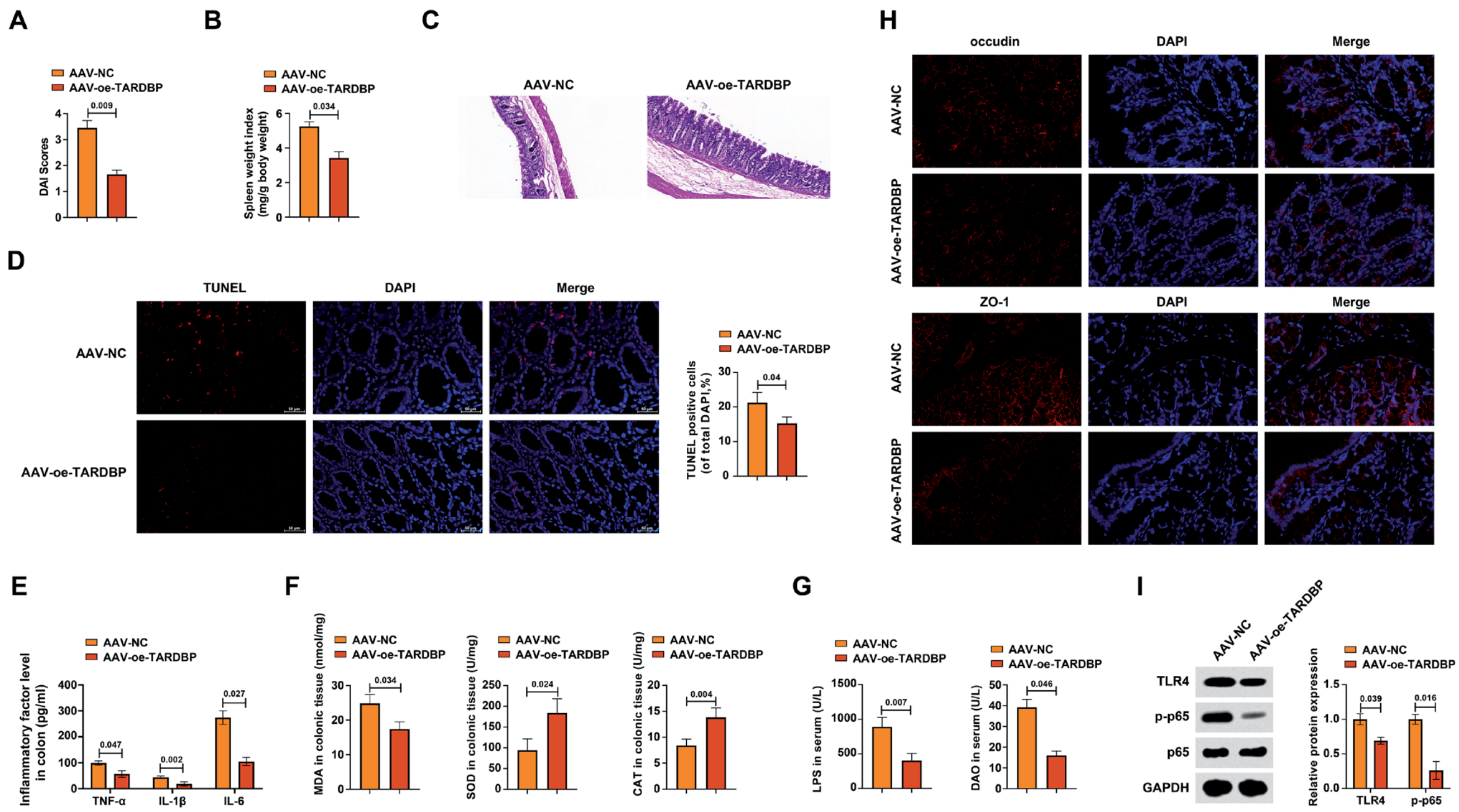

Supplementary Figure 6. TARDBP overexpression protects against DSS-induced colonic injury. (A) Time-dependent effects of DSS treatment on functional impairment of the colon in mice were assessed by calculating DAI. (B) Effects of DSS treatment on systemic immune activation in mice were assessed by measuring the spleen weights. (C) Histological structure of epithelial cells of the colon of mice was assessed by HE staining. (D) Changes of apoptosis rate in the colon of mice were detected by TUNEL staining. (E) Levels of inflammatory factors TNF-α, IL-1β and IL-6 were detected by ELISA in the colon tissues of mice. (F) Oxidative damage was assessed by detecting the oxidative stress indicators MDA, SOD and CAT in the colon tissues of mice. (G) Intestinal permeability was assessed by serum LPS and DAO analysis in mice. (H) Changes in the expression of ZO-1 and occludin proteins in the colon tissues of mice were detected by IF staining. (I) Changes in TLR4 and p-p65 protein expression in the colon tissues of mice were detected by Western blot. Data are expressed as mean ± SD (n = 8). *P < 0.05.

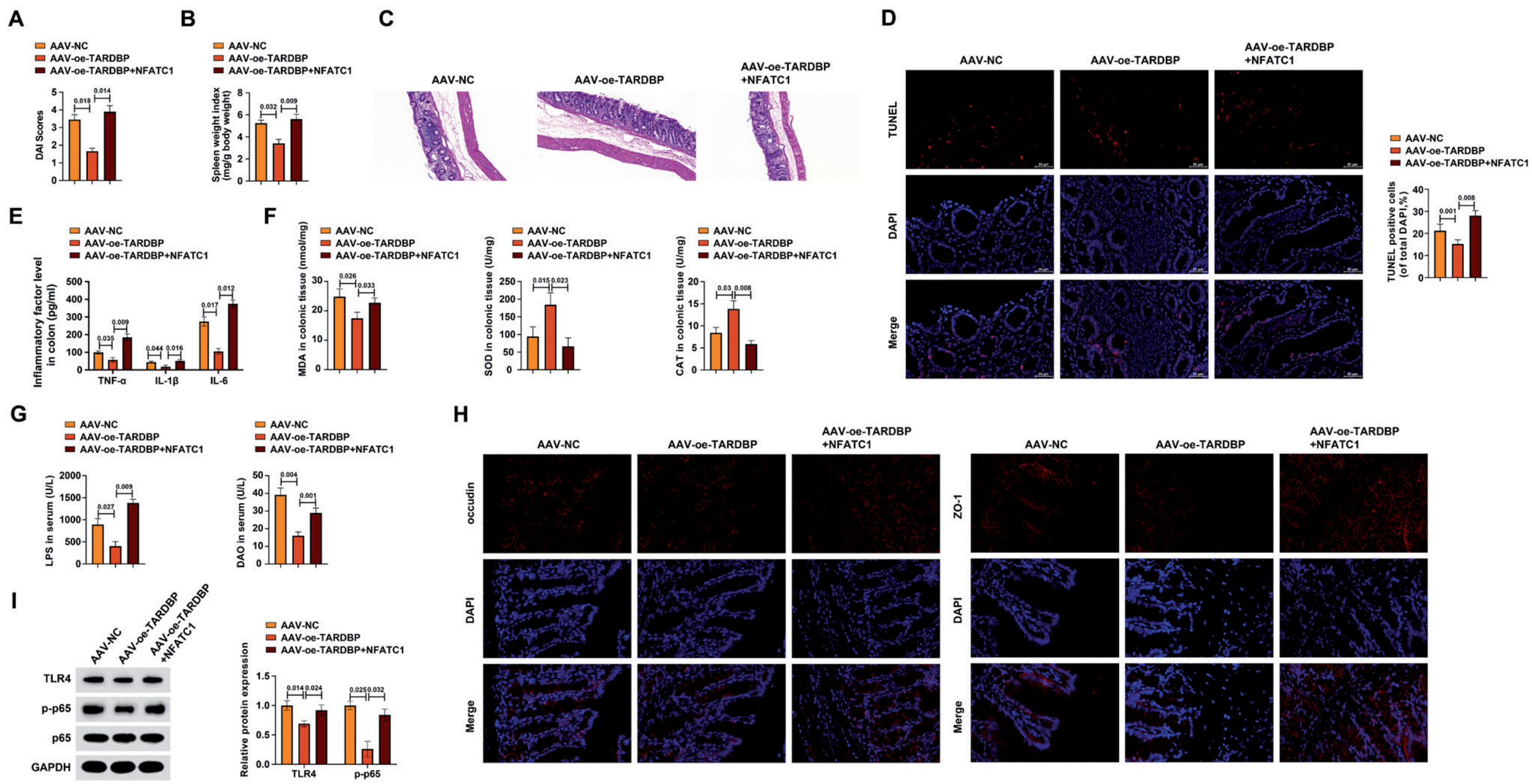

Supplementary Figure 7. NFATC1 overexpression reverses the protective effects of TARDBP. (A) Time-dependent effects of DSS treatment on functional impairment of the colon in mice were assessed by calculating DAI. (B) Effects of DSS treatment on systemic immune activation in mice were assessed by measuring the spleen weights. (C) Histological structure of epithelial cells of the colon of mice was assessed by HE staining. (D) Changes of apoptosis rate in the colon of mice were detected by TUNEL staining. (E) Levels of inflammatory factors TNF-α, IL-1β and IL-6 were detected by ELISA in the colon tissues of mice. (F) Oxidative damage was assessed by detecting the oxidative stress indicators MDA, SOD and CAT in the colon tissues of mice. (G) Intestinal permeability was assessed by serum LPS and DAO analysis in mice. (H) Changes in the expression of ZO-1 and occludin proteins in the colon tissues of mice were detected by IF staining. (I) Changes in TLR4 and p-p65 protein expression in the colon tissues of mice were detected by Western blot. Data are expressed as mean ± SD (n = 8). *$P < 0.05$.
